# Radiomics-Based AI Model to Assist Clinicians in Intracranial Hemorrhage Diagnosis: External Validation Study

**DOI:** 10.2196/81038

**Published:** 2025-12-11

**Authors:** Salita Angkurawaranon, Natipat Jitmahawong, Kittisak Unsrisong, Phattanun Thabarsa, Chakri Madla, Withawat Vuthiwong, Thanwa Sudsang, Chaisiri Angkurawaranon, Patrinee Traisathit, Papangkorn Inkeaw

**Affiliations:** 1 Department of Radiology, Faculty of Medicine, Chiang Mai University, Chiang Mai, Thailand; 2 Global Health and Chronic Conditions Research Group, Chiang Mai University, Chiang Mai, Thailand; 3 Master’s Degree Program in Data Science, Faculty of Engineering, Chiang Mai University, Chiang Mai, Thailand; 4 Department of Radiology, Ramathibodi Hospital, Mahidol University, Bangkok, Thailand; 5 Department of Family Medicine, Faculty of Medicine, Chiang Mai University, Chiang Mai, Thailand; 6 Department of Statistics, Faculty of Science, Chiang Mai University, Chiang Mai, Thailand; 7 Data Science Research Center, Faculty of Science, Chiang Mai University, 239 Huay Kaew Road, Muang District, Chiang Mai, 50200, Thailand, 66 903327931; 8 Department of Computer Science, Faculty of Science, Chiang Mai University, Chiang Mai, Thailand

**Keywords:** radiomics, spontaneous intracranial hemorrhages, external validation, artificial intelligence, AI, AI-assisted decision-making, brain computed tomography scans, brain CT scans

## Abstract

**Background:**

Early identification of the etiology of spontaneous intracerebral hemorrhage (ICH) could significantly contribute to planning a suitable treatment strategy. A notable radiomics-based artificial intelligence (AI) model for classifying causes of spontaneous ICH from brain computed tomography scans has been previously proposed.

**Objective:**

This study aimed to externally validate and assess the utility of this AI model.

**Methods:**

This study used 69 computed tomography scans from a separate cohort to evaluate the AI model’s performance in classifying nontraumatic ICHs into primary, tumorous, and vascular malformation related. We also assessed the accuracy, sensitivity, specificity, and positive predictive value of clinicians, radiologists, and trainees in identifying the ICH causes before and after using the model’s assistance. The performances were statistically analyzed by specialty and expertise levels.

**Results:**

The AI model achieved an overall accuracy of 0.65 in classifying the 3 causes of ICH. The model’s assistance improved overall diagnostic performance, narrowing the gap between nonradiology and radiology groups, as well as between trainees and experts. The accuracy increased from 0.68 to 0.72, from 0.72 to 0.76, from 0.69 to 0.74, and from 0.72 to 0.75 for nonradiologists, radiologists, trainees, and specialists, respectively. With the model’s support, radiology professionals demonstrated the highest accuracy, highlighting the model’s potential to enhance diagnostic consistency across different levels.

**Conclusions:**

When applied to an external dataset, the accuracy of the AI model in categorizing spontaneous ICHs based on radiomics decreased. However, using the model as an assistant substantially improved the performance of all reader groups, including trainees and radiology and nonradiology specialists.

## Introduction

Spontaneous intracerebral hemorrhage (ICH) accounts for 10% to 15% of all strokes and is associated with a higher mortality rate than either ischemic stroke or subarachnoid hemorrhage (SAH). The causes of ICH are divided into primary and secondary. Primary causes of nontraumatic ICH associated with chronic hypertension or amyloid angiopathy and secondary causes associated with vascular abnormalities or neoplasms are among the most prevalent [1]. The principal treatments for primary ICH are surgical hematoma excision and conservative therapy [2]. Secondary ICH requires further attention to address the underlying disease. Subsequently, the early identification of the etiology of spontaneous ICH can significantly contribute to the optimization of the prognosis of individual patients and the planning of a suitable treatment strategy [3].

Computed tomography (CT) is typically the initial imaging modality for diagnosing and determining the cause of ICH. However, non–contrast-enhanced CT scans often fail to reliably identify the underlying etiology of spontaneous ICH [3,4]. A hemorrhage associated with secondary causes of ICH, especially tumors and vascular malformations, can be masked and lead to a misdiagnosis [5-7]. Misclassification of the causes of ICH can lead to delayed diagnoses and worsen patient outcomes.

Radiomics, a rapidly advancing field, uses advanced image analysis to extract quantitative features that extend beyond what is visible to the human eye [8]. By applying mathematical approaches, radiomics enables the quantification of visual attributes, offering deeper insights into imaging data. There has been growing interest in deploying machine learning in clinical practice in recent years. Rather than replacing radiologists and clinicians, machine learning models are increasingly recognized as tools that support and enhance decision-making processes [9-11]. One area that remains underexplored is the potential of these models to improve radiologists’ and clinicians’ diagnostic performance through collaborative decision-making between humans and artificial intelligence (AI) models. This potential is particularly significant given the global shortage of radiologists and the increasing volume of imaging studies [12]. Previous studies have combined radiomics with machine learning models to differentiate between various causes of nontraumatic ICH, including vascular malformation–related hemorrhage versus primary ICH [13], arteriovenous malformation–related hematomas versus other spontaneous ICHs [14], primary versus secondary ICH [15], and neoplastic versus nonneoplastic ICH [16].

A notable study by Thabarsa et al [17] used radiomics and machine learning to classify the 3 most common causes of nontraumatic ICH—primary, tumorous, and vascular malformation–related hemorrhages—from brain CT scans. This multiclass approach aligns more closely with real-world clinical practice than previous studies that classified just 2 causes of spontaneous ICHs [13-16]. The aforementioned study also differs from other radiomics studies that exclusively concentrate on the hematoma region or the edema region [13,14]. Thabarsa et al [17] applied radiomics features from both regions, with the results demonstrating that, when features are chosen from both regions, this improves the classification performance of the model [16]. Although the sensitivity and area under the curve were marginally lower than in previous research, the specificity was higher, indicating a satisfactory performance [13,15]. However, a critical limitation of prior studies is lack of external validation, which hampers the generalizability of radiomics-based prediction models and their integration into clinical workflows [13-17].

The aim of this study was to externally evaluate radiomics-based models to classify causes of nontraumatic ICH from CT scans of the brain on an independent cohort. Furthermore, this study evaluated the performance of trainee clinicians and trainee radiologists and of clinicians and radiologists with and without the support of a machine learning model.

## Methods

### Overview

**Figure 1.**
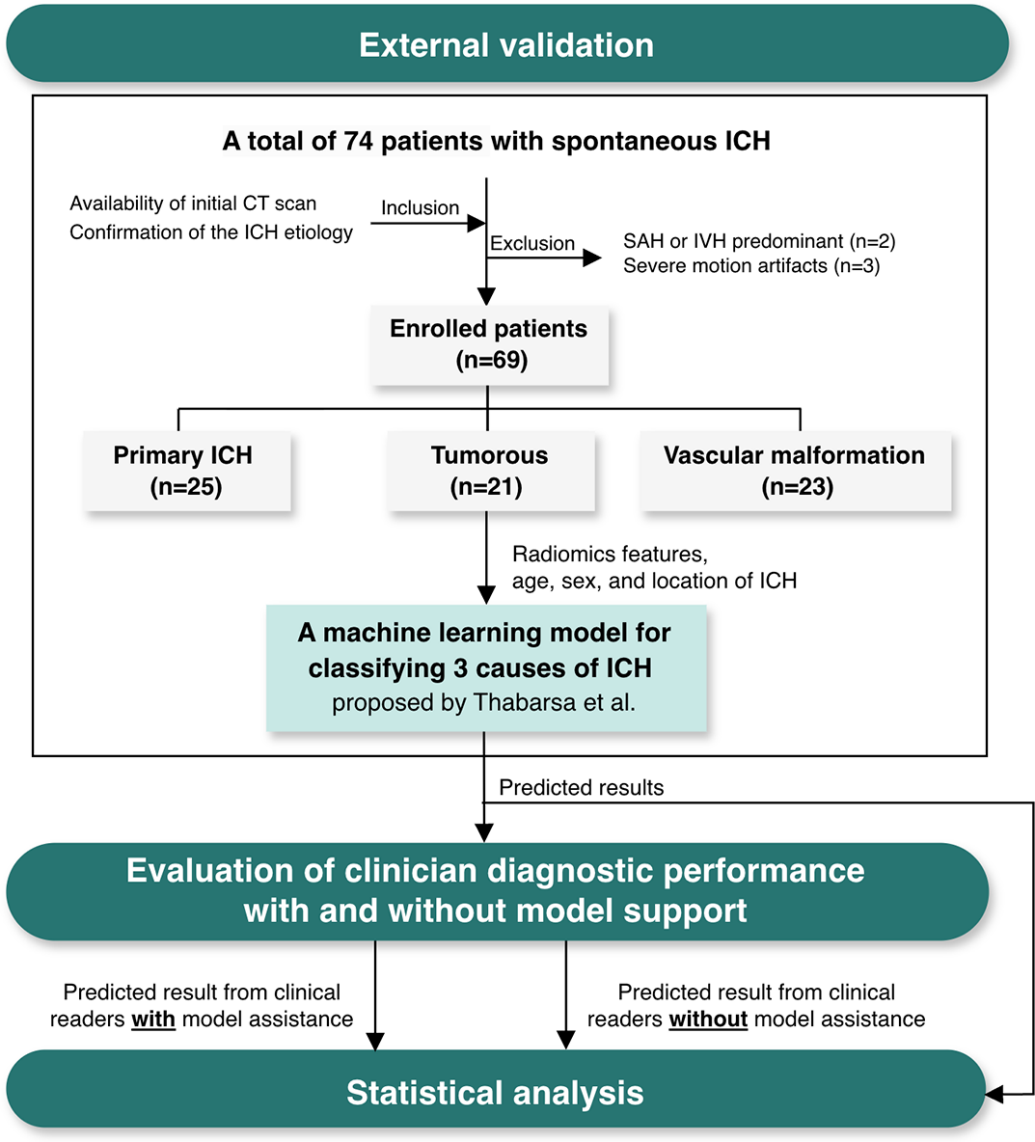
The workflow of this study [17]. CT: computed tomography; ICH: intracerebral hemorrhage; IVH: intraventricular hemorrhage; SAH: subarachnoid hemorrhage.

This study used a previously published machine learning model to classify the etiologies of nontraumatic ICH into primary, tumorous, and vascular malformation related [17]. The model was first evaluated using an external validation dataset. It was then assessed for its potential to enhance clinicians’ diagnostic performance. An overview of the study is shown in Figure 1 [17].

### Ethical Considerations

The Ethics Committee of the Faculty of Medicine Ramathibodi Hospital, Mahidol University, Thailand, granted permission for the acquisition of the external validation dataset from Ramathibodi Hospital (approval COA.MURA2022/431; approval date: August 3, 2022). As the study was retrospective in nature and all procedures were part of standard clinical care, the committee waived the informed consent requirement. To maintain privacy and confidentiality, all data were completely anonymized before analysis. This study did not involve participant compensation.

### External Validation Data

The dataset used in this study was obtained from Ramathibodi Hospital. We conducted a retrospective evaluation of the medical reports of patients who experienced spontaneous ICH from the radiology department database between January 2010 and January 2020. Data collected included noncontrast CT (NCCT) brain scans, as well as patient age, sex, and ICH location. Inclusion criteria were as follows: (1) availability of initial CT scan after onset of symptoms and (2) confirmation of the ICH etiology through follow-up magnetic resonance imaging, pathological reports, or additional digital subtraction angiography for primary, tumorous, and vascular malformation–related ICH. Exclusion criteria included hematomas with SAH or intraventricular hemorrhage predominant. Low-quality NCCT images with severe artifacts, such as metallic or motion artifacts, were also excluded.

Of the 74 NCCT brain scans reviewed, 69 (93.2%) were included in the study. The remaining 6.8% (5/74) of the scans were excluded due to severe artifacts (n=3) or the presence of hematomas with SAH or intraventricular hemorrhage predominant (n=2). The scans were first anonymized and set to a brain window (level: 80 HU; width: 40 HU), slice thickness of 2.5 to 5 mm, and image resolution of 512 × 512 pixels. The CT scans were performed using 1 of the 4 institute machines: a GE Revolution Frontier spectral CT scanner, a GE Revolution spectral CT scanner, a Philips IQon spectral CT scanner, and a Philips Brilliance iCT scanner. Data on age, sex, and ICH location were also obtained. The ICH location was classified into deep, lobar, and infratentorial regions. The study by Thabarsa et al [17] provides a detailed explanation of these classifications, as well as the process of feature extraction for hemorrhages and the vasogenic edema that surrounds them.

### Machine Learning Model for Classifying Causes of ICH

In this study, we adopted a radiomics-based machine learning model proposed by Thabarsa et al [17] for classifying causes of ICHs. The model was organized in a hierarchical structure that consists of 2 adaptive boosting classifiers. First, it discriminates between tumor and nontumor causes of ICH through the first classifier. If the case is classified as nontumor, a second classifier is applied to further differentiate between primary and vascular malformation–related ICH. The first classifier uses radiomics features extracted from both the hematoma and edema regions, including 2 first-order, 2 gray-level co-occurrence matrix (GLCM), and 3 gray-level dependence matrix (GLDM) features from the hematoma region and 1 GLDM, 1 gray-level run-length matrix, 2 gray-level size-zone matrix (GLSZM), and 3 GLCM features from the edema region. Meanwhile, the second classifier uses 10 radiomics features extracted from the hematoma (ie, 2 GLDM, 2 GLCM, and 1 GLSZM) and edema (ie, 2 GLDM, 1 GLCM, 1 GLSZM, and 1 gray-level run-length matrix) regions as predictors. Furthermore, age and ICH location were incorporated as supplementary predictors for classifying the causes of ICH in both classifiers.

The model was trained and tested on 141 NCCT scans using 5-fold cross-validation. All scans were collected from Maharaj Nakorn Chiang Mai Hospital. Specifically, of the 141 scans, 26 (18.4%) were obtained from a Toshiba Aquilion 16-slice CT scanner, 89 (63.1%) were obtained from a 64-slice Siemens Definition multidetector CT (Germany) scanner, and 26 (18.4%) were obtained from a 192-slice Siemens Definition dual-source CT (Germany) scanner. The scans were acquired using a brain window (level: 80 HU; width: 40 HU), with a slice thickness of 5 mm and an image resolution of 512 × 512 pixels.

### Clinicians’ Performance With AI Assistance

To assess the potential of the proposed machine learning model as a diagnostic assistance tool in distinguishing the 3 causes of ICHs, clinical readers from diverse specialties and varying levels of expertise participated. They included 4 general radiologists, 8 radiology residents, 2 emergency medicine physicians, 2 emergency medicine residents, 2 neurosurgeons, and 2 neurosurgery residents. Their performance was compared with and without model assistance and analyzed by specialty and level of expertise.

**Figure 2.**
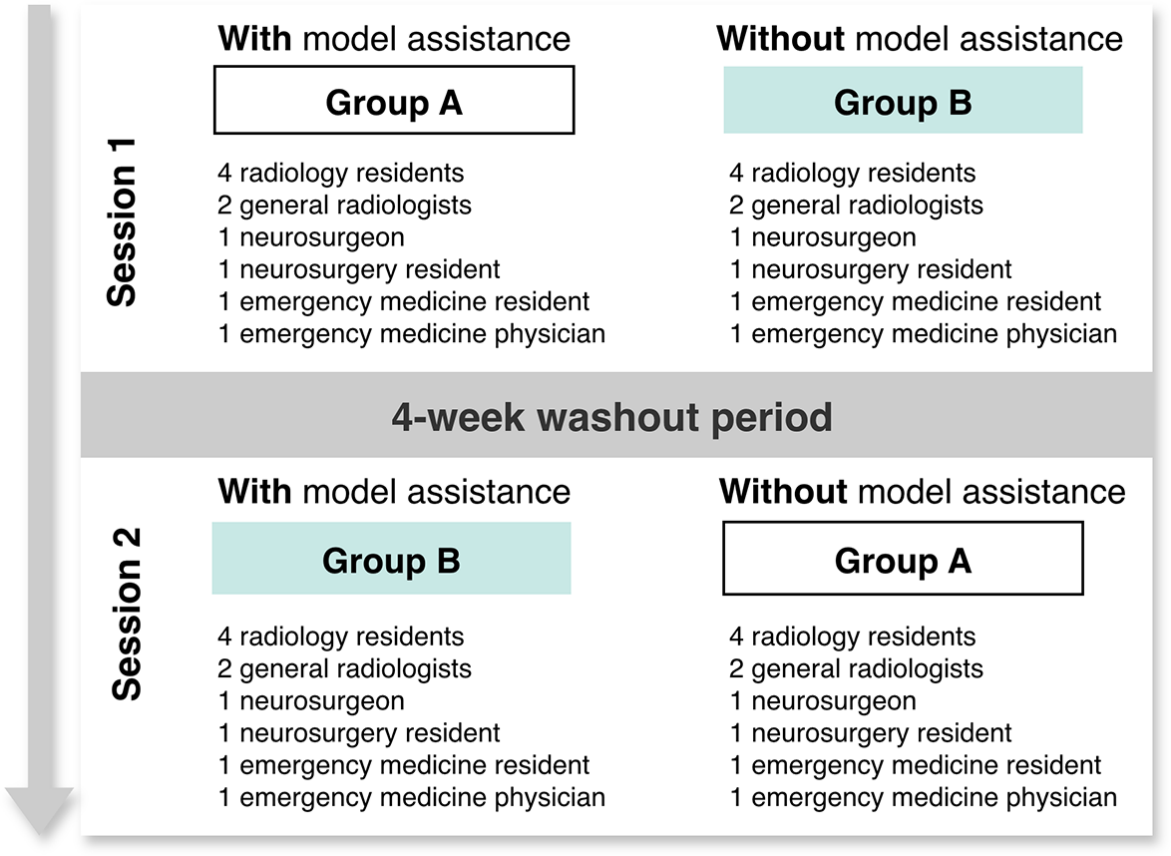
Study design for evaluation of clinician diagnostic performance with and without model support.

The readers were divided into 2 groups (group A and group B). In the first session, group A interpreted the CT scans with model assistance, whereas group B interpreted them without model assistance. Following the session, a 4-week washout period took place, during which the groups were reshuffled to minimize familiarization before the next session. In the second session, group A interpreted the CT scans without model assistance, whereas group B used model assistance. Patient age and sex information was also made available to all readers. In both sessions, each reader was assigned a unique, nonrepeating order of patients (Figure 2).

### Statistical Analysis

Demographic and clinical variables between external validation datasets were compared using the Kruskal-Wallis test for continuous variables and the Fisher exact test for categorical variables. To investigate the effects of CT scan manufacturers and slice thickness on predictive error, a chi-square test was also used. In addition, we investigated the differences in the diagnostic performances of nonradiologists and radiologists before and after adopting the machine learning model’s assistance. The nonradiology readers included trainees and clinicians in emergency medicine and neurosurgery, whereas the radiology readers consisted of radiology residents and radiologists. In addition, we explored improvements in the diagnostic ability to identify ICH causes in groups of trainees and specialists after being assisted by the machine learning model. Residents from the radiology, neurosurgery, and emergency medicine departments were among the trainee readers. Radiologists, neurosurgeons, and emergency medicine physicians were grouped as specialty readers. Significant differences measured using the Friedman test were considered at *P*<.05. The diagnostic performance was evaluated using accuracy, sensitivity, specificity, and positive predictive value (PPV), with the analysis conducted using R (version 4.3.2; R Foundation for Statistical Computing).

## Results

### Study Population 

On the basis of the participant flowchart (Figure 1), 69 patients met the eligibility criteria and were enrolled in the study. A summary of the patient demographics is provided in Table 1. Age and ICH location showed no significant differences in the underlying causes of ICH (*P*=.81 and *P*=.41, respectively). Sex, which was not considered in the diagnostic process of the model, also showed no significant differences across the classes (*P*=.16).

**Table 1.** Patient demographic and clinical characteristics of the external validation dataset (N=69).

Variable	Patients with spontaneous intracerebral hemorrhage			*P* value
	Primary (n=25)	Tumorous (n=21)	Vascular malformation related (n=23)	
Age (y), mean (SD)	51.5 (21.8)	55.8 (18.1)	43.2 (23.0)	.81
Sex, n (%)				.16
Male	14 (56)	13 (61.9)	12 (52.2)	
Female	11 (44)	8 (38.1)	11 (47.8)	
Location, n (%)				.41
Deep	6 (24)	2 (9.5)	3 (13)	
Infratentorial	1 (4)	0 (0)	0 (0)	
Lobar	18 (72)	19 (90.5)	20 (87)	

### Model Performance

The machine learning model achieved an overall accuracy of 0.65 in the classification of the 3 causes of ICH. The model performance metrics for each cause of ICH on the external dataset are shown in Table 2. For primary ICH, the sensitivity, specificity, and PPV were 0.64, 0.82, and 0.67, respectively. For tumor-related ICH, the model achieved sensitivity, specificity, and PPV of 0.81, 0.79, and 0.63. The sensitivity, specificity, and PPV for vascular malformation–related ICH were 0.52, 0.87, and 0.66, respectively.

**Table 2.** Classification performance of the machine learning model on the external validation dataset.

Cause of ICH^a^	Performance metric			
	Sensitivity	Specificity	PPV^b^	AUC^c^
Primary	0.64	0.82	0.67	0.59
Tumorous	0.81	0.79	0.63	0.84
Vascular malformation	0.52	0.87	0.67	0.65

a ICH: intracerebral hemorrhage.

b PPV: positive predictive value.

c AUC: area under the curve.

We further analyzed the effects of CT scan manufacturers and slice thickness on predictive error using a chi-square test. The results showed that, for primary ICH, no statistically significant association was observed between slice thickness and prediction accuracy (*P*=.11). Similarly, the manufacturer was not significantly associated with prediction accuracy (*P*=.77). For tumor-related ICH, slice thickness showed no statistically significant relationship with prediction accuracy (*P*=.50), and the manufacturer also showed no significant association (*P*=.66). For vascular malformation–related ICH, there was no statistically significant association between slice thickness and prediction accuracy (*P*=.41). Similarly, the manufacturer was not significantly associated with prediction accuracy (*P*=.74).

### Evaluation of Clinician Diagnostic Performance With and Without AI Support

#### Diagnostic Performance of Readers With and Without AI Support by Clinical Specialty

Table 3 presents the diagnostic performance of readers in identifying causes of acute ICH both with and without the assistance of AI. The readers were categorized by clinical specialty as nonradiology and radiology readers. As can be observed from the results, radiology readers consistently outperformed nonradiology readers in both situations. The model was able to assist both nonradiology and radiology readers in identifying the cause of ICH by substantially improving the overall accuracies from 0.68 to 0.72 and from 0.72 to 0.76, respectively. The sensitivity, specificity, and PPV for primary, tumorous, and vascular malformation–related ICH increased in both reader groups after being assisted by the model. The sensitivity, specificity, and PPV in radiologists significantly improved by 0.09, 0.07, and 0.16, respectively, especially for identifying the vascular etiology of ICH. The PPV in nonradiologists also increased by 0.11.

**Table 3.** Diagnostic performance of readers with and without artificial intelligence support by clinical specialty.

Cause of acute ICH^a^ and performance metric	Nonradiology (n=8)			Radiology (n=12)		
	Without model assistance	With model assistance	*P* value	Without model assistance	With model assistance	*P* value
Overall						
Accuracy (95% CI)	0.68 (0.65-0.71)	0.72 (0.70-0.74)	.02^b^	0.72 (0.70-0.74)	0.76 (0.74-0.78)	.003^b^
Primary						
Sensitivity (95% CI)	0.43 (0.32-0.53)	0.50 (0.40-0.59)	.31	0.49 (0.43-0.55)	0.57 (0.50-0.64)	.20
Specificity (95% CI)	0.81 (0.75-0.87)	0.80 (0.74-0.86)	.74	0.84 (0.79-0.88)	0.85 (0.82-0.88)	.66
PPV^c^ (95% CI)	0.58 (0.53-0.63)	0.59 (0.55-0.63)	.84	0.65 (0.58-0.71)	0.68 (0.65-0.72)	.27
Tumorous						
Sensitivity (95% CI)	0.75 (0.62-0.88)	0.82 (0.76-0.88)	.31	0.87 (0.82-0.91)	0.88 (0.84-0.91)	.57
Specificity (95% CI)	0.75 (0.66-0.84)	0.79 (0.74-0.84)	.31	0.76 (0.71-0.80)	0.77 (0.75-0.79)	.79
PPV (95% CI)	0.58 (0.51-0.65)	0.64 (0.59-0.69)	.12	0.62 (0.58-0.65)	0.63 (0.61-0.64)	.57
Vascular malformation						
Sensitivity (95% CI)	0.42 (0.36-0.48)	0.47 (0.42-0.51)	.20	0.41 (0.34-0.48)	0.50 (0.45-0.54)	.01^b^
Specificity (95% CI)	0.72 (0.66-0.78)	0.80 (0.75-0.83)	.06	0.78 (0.75-0.81)	0.85 (0.81-0.88)	.02^b^
PPV (95% CI)	0.44 (0.38-0.49)	0.55 (0.49-0.58)	.04^b^	0.47 (0.42-0.53)	0.63 (0.57-0.69)	.005^b^

a ICH: intracerebral hemorrhage.

b Statistically significant difference in performance between clinicians with and without model assistance.

c PPV: positive predictive value.

#### Diagnostic Performance of Readers With and Without AI Support by Level of Expertise

Table 4 compares the diagnostic performance of readers, grouped as trainees and specialists, in identifying causes of acute ICH with and without the assistance of AI. The specialists outperformed the trainees in both nonassistance and assistance situations. After adopting the model, trainee and specialist readers improved significantly in determining what caused ICH. The overall accuracies increased from 0.69 to 0.74 for trainees and from 0.72 to 0.75 for specialists. For primary causes, the assistance of the AI model improved diagnostic performance in both reader groups. The sensitivity for trainees significantly increased from 0.46 to 0.54. After the model assisted in identifying tumor causes of ICH, all performance metrics for both trainees and specialists increased by approximately 0.01 and 0.07, except for sensitivity for specialists, which slightly dropped by 0.02. Interestingly, the assistance from the model was able to enhance the diagnostic performance for identifying vascular causes of ICH. The sensitivity, specificity, and PPV in the trainee group significantly increased from 0.41, 0.73, and 0.43 to 0.49, 0.73, and 0.59, respectively. For specialists, these performance measurements also improved: the sensitivity slightly increased from 0.42 to 0.49, whereas the specificity and PPV significantly improved from 0.79 and 0.50 to 0.84 and 0.62, respectively.

**Table 4.** Diagnostic performance of readers with and without artificial intelligence (AI) support by level of expertise.

Cause of acute ICH^a^ and performance metric	Trainees (n=12)			Specialists (n=8)		
	Without AI assistance	With AI assistance	*P* value	Without AI assistance	With AI assistance	*P* value
Overall						
Accuracy (95% CI)	0.69 (0.67-0.72)	0.74 (0.73-0.76)	.003^b^	0.72 (0.68-0.75)	0.75 (0.72-0.78)	.02^b^
Primary						
Sensitivity (95% CI)	0.46 (0.40-0.52)	0.54 (0.48-0.61)	.045^b^	0.47 (0.36-0.58)	0.54 (0.42-0.65)	.06
Specificity (95% CI)	0.81 (0.77-0.86)	0.83 (0.79-0.86)	.55	0.84 (0.78-0.90)	0.83 (0.77-0.88)	.18
PPV^c^ (95% CI)	0.60 (0.54-0.67)	0.65 (0.60-0.70)	.17	0.64 (0.58-0.71)	0.64 (0.60-0.69)	.93
Tumorous						
Sensitivity (95% CI)	0.78 (0.69-0.88)	0.85 (0.81-0.89)	.09	0.88 (0.83-0.92)	0.86 (0.81-0.92)	.67
Specificity (95% CI)	0.77 (0.72-0.81)	0.79 (0.76-0.82)	.37	0.74 (0.65-0.82)	0.76 (0.73-0.80)	.38
PPV (95% CI)	0.60 (0.55-0.64)	0.64 (0.61-0.67)	.14	0.61 (0.55-0.67)	0.62 (0.59-0.64)	.31
Vascular malformation						
Sensitivity (95% CI)	0.41 (0.34-0.47)	0.49 (0.44-0.53)	.04^b^	0.42 (0.35-0.49)	0.49 (0.44-0.53)	.06
Specificity (95% CI)	0.73 (0.69-0.77)	0.82 (0.78-0.85)	.02^b^	0.79 (0.75-0.83)	0.84 (0.79-0.89)	.02^b^
PPV (95% CI)	0.43 (0.39-0.48)	0.59 (0.52-0.63)	.009^b^	0.50 (0.44-0.56)	0.62 (0.54-0.70)	.02^b^

a ICH: intracerebral hemorrhage.

b Statistically significant difference in performance between clinicians with and without model assistance.

c PPV: positive predictive value.

## Discussion

As is common in external validation studies, the performance of the model by Thabarsa et al [17] on external data showed decreased accuracy compared to its performance on internal data by approximately 17.7%, from 0.79 to 0.65. However, the performance of readers, including trainees, clinicians, and radiologists, improved after using AI-assisted interpretations of the causes of spontaneous ICH.

### External Validation of Model Performance

The lack of emphasis on external validation as a critical factor in assessing the clinical utility of AI models is reflected in the fact that only 6% to 13% of AI papers in medical imaging include external validation [18-20]. This oversight limits the understanding of these models’ true potential in real-world applications. Our study addresses this critical gap by demonstrating that the machine learning model by Thabarsa et al [17] exhibited inferior performance on external datasets compared to its performance on internal data. This finding highlights the challenges of generalizability and underscores the necessity of testing AI models on diverse, independent datasets before clinical deployment. Additionally, our results are consistent with those of a systematic review of the external validation of deep learning models for radiologic diagnosis [21]. In that review, it was discovered that 81% of the studies experienced a decline in performance as a result of the use of external datasets. The extent of the decline varied from a slight decrease to a 24% decrease.

The precise reasons for the reduced performance of machine learning algorithms using external datasets remain mostly unidentified. Our study’s external dataset revealed no significant differences in ICH locations and patient age among the various causes of ICH. These characteristics were incorporated into the initial machine learning model in conjunction with radiomics features as they enhanced the model’s performance. These variations in data characteristics may have contributed to the model’s diminished performance when applied to external data. Different CT machines provided the training data and external validation data for our investigation. The thickness of the CT scan was also different between the different scanners. Although a previous study reported that the differences in scanners, imaging techniques or protocols, and reconstruction parameters can substantially affect the stability and reproducibility of radiomics features [22], in this study, the difference in CT scan manufacturers did not affect the predictive error. In addition, the different settings of slice thickness were not significantly related to the predictive error. The reduced performance in the external validation was mainly due to variations in population characteristics between the external validation sample and the training dataset.

Before clinical deployment, it is recommended that the model be validated using independent data that share similar characteristics with the data expected in the intended deployment setting. As the model provides a prediction with a confidence score, the score should be considered and used for decision-making. Furthermore, explainable AI techniques such as Shapley additive explanations and local interpretable model-agnostic explanations can be adopted to understand how the model makes decisions for an input sample. Shapley additive explanations compute the contribution scores of each feature to the prediction. Meanwhile, local interpretable model-agnostic explanations explain the individual predictions of any machine learning model by approximating its behavior using a simpler, interpretable model (eg, linear model and decision tree) in the local region of the prediction. The explainable AI techniques provide useful information that radiologists could consider before deciding. In practical use, when a radiologist’s impression differs from the model’s prediction, especially when the model indicates a hemorrhage, the model’s output may serve as a prompt to broaden the differential diagnosis. This could encourage further investigation, such as vascular imaging or follow-up studies, to reach a definite diagnosis. Although the model does not provide voxel-level explanations, its prediction can still function as a clinically meaningful cue that supports radiologists in considering alternative etiologies.

### Clinician Diagnostic Performance With and Without AI Support

A prior study assessed the effectiveness of a machine learning model using radiomics features derived from CT scans in distinguishing arteriovenous malformation–related ICHs from those resulting from other causes compared to radiologists’ evaluations [14]. The outcomes showed that the model outperformed radiologists. Nonetheless, the primary function of machine or deep learning models is to assist radiologists and radiology trainees rather than replace them in clinical environments. AI systems’ decision-making procedures lack openness compared to radiologists’ clinical interpretation due to their “black box” design [23]. As a consequence, our investigation assessed the performance of trainees and specialty readers both with and without consideration of the results of the AI model. Without the assistance of the AI model, the nonradiology group demonstrated a lower performance than that of the radiology group, and trainees lagged behind specialists. However, our findings revealed that the use of AI improved reader performance, narrowing the gap between these groups. The model significantly improved the performance of all reader groups in predicting vascular malformations as the cause of spontaneous ICH. Notably, AI assistance had the greatest impact on nonradiology readers and trainees, elevating their performance to levels comparable to that of radiologists or specialists working without AI support.

This model has potential value in the context of real-world medicine. Most emergency radiology units, including that in our hospital, depend on radiology residents to perform some of their nighttime duties [24]. Radiology residents’ work is regarded as a critical component of their education as it not only minimizes the burden of the attending radiologists but also improves the abilities of the residents. However, studies have shown that diagnostic error rates tend to increase during night shifts, particularly after midnight, likely due to fatigue and reduced alertness [25]. This AI model could support nighttime shifts, which could reduce diagnostic errors while maintaining the educational benefits of resident training. Moreover, while using the model, the performance of groups of professionals and radiology readers also showed the best results. Meanwhile, radiology residency programs should foster independent reasoning, critical evaluation, and comprehensive understanding of AI principles [26]. To prevent the integration of AI from undermining residents’ skill development, maintaining a balanced exposure to both AI-assisted and independent case interpretation is crucial. Regular comparison of performance, analysis of discrepancies between AI and human interpretations, and reflective discussion can help sustain residents’ analytical autonomy while supporting the responsible incorporation of AI into radiology education.

AI-assisted data interpretation significantly improved all readers’ ability to correctly identify vascular malformation causes. Unlike primary ICH, vascular malformation–related ICH demonstrates greater complexity and heterogeneity in internal composition and morphology due to the presence of malformed blood vessels, as reflected in radiomics features [13]. This finding aligns with those of previous research showing that radiomics features can effectively differentiate vascular malformations from other causes of ICH [13,14]. AI support also improves the capacity of clinicians to predict primary and tumor-related ICH but does not achieve statistical significance.

There are several limitations to this study. First, the relatively small sample size and retrospective design may limit the robustness and reproducibility of the findings. The small sample size likely resulted in low statistical power. It may not yield statistically significant results even if the model has some predictive ability. However, our study also attempted to incorporate external data, a topic on which there is currently limited research for machine learning models with radiomics features. Larger studies or additional external datasets would be useful for future studies. Second, the manual segmentation process used was time-consuming and subjective and introduced inter- and intra-annotator variability, potentially biasing the model’s performance. Third, the model was developed using data from a single institution and externally validated on data from only 1 additional hospital, which may limit its generalizability to other centers with different scanners, imaging protocols, or patient populations. Future research could focus on prospective validation and on exploring the role of AI in enabling fast and accurate segmentation [27,28], which would help standardization and translation of radiomics into real-world radiology.

### Conclusions

This study’s findings demonstrated that the machine learning model by Thabarsa et al [17] for categorizing spontaneous ICHs using radiomics characteristics exhibited reduced performance when applied to an external dataset. Nevertheless, the model significantly enhanced the performance of all reader groups, including trainees and both radiology and nonradiology specialists, when used as a supportive tool.

## Abbreviations

AI: artificial intelligence
CT: computed tomography
GLCM: gray-level co-occurrence matrix
GLDM: gray-level dependence matrix
GLSZM: gray-level size-zone matrix
ICH: intracerebral hemorrhage
NCCT: noncontrast computed tomography
PPV: positive predictive value
SAH: subarachnoid hemorrhage
